# Menstrual Hygiene Management Practices and Associated Factors Among Female High School Students in Awsi Resu, Afar Region, Ethiopia: A Cross‐Sectional Study

**DOI:** 10.1002/hsr2.72108

**Published:** 2026-03-15

**Authors:** Salahedin Getahun, Mohammed Abdu, Frewein Yilma, Adem Yesuf, Ali Yimer, Hassen Ahmed Yesuf, Bewuketu Belete Alemu, Alemken Eyayu Abuhay, Degsew Ewunetie Anteneh

**Affiliations:** ^1^ Department of Public Health, College of Medicine and Health Sciences Samara University Samara Ethiopia; ^2^ Department of Nursing and Midwifery College of Health Sciences, Woldia University Woldia Ethiopia; ^3^ Department of Public Health College of Health Sciences, Woldia University Woldia Ethiopia; ^4^ Department of Medical Laboratory College of Medicine and Health Science, Woldia University Woldia Ethiopia; ^5^ Department of Clinical Midwifery University of Gondar Comprehensive Specialized Hospital Gondar Ethiopia; ^6^ Department of Midwifery College of Medicine and Health Science, Woldia University Woldia Ethiopia

**Keywords:** Ethiopia, female students, menstrual hygiene practice

## Abstract

**Background:**

Poor menstrual hygiene management (MHM), characterized by inadequate use of clean materials, improper disposal, and poor hygiene practices, negatively impacts the health of reproductive‐age women. In school settings, these practices further compromise girls' health, well‐being, and educational performance. This study assessed menstrual hygiene management practices and associated factors among female high school students in Awsi Rasu, Afar Region, Northeast Ethiopia, 2023.

**Methods:**

An institutional‐based cross‐sectional study was conducted from April 15 to 30, 2023, among 423 female high school students in the Afar Region, Ethiopia. Data were collected using self‐administered structured questionnaires. Participants were selected through a simple random sampling technique. The collected data were coded and entered into EpiData version 4.6 and then exported to SPSS version 25.0 for analysis. Descriptive statistics were employed to assess menstrual hygiene management practices, while binary logistic regression was used to identify associated factors. Statistical significance was determined at a *p*‐value of < 0.05.

**Result:**

This study found that 51.3% (95% CI: 46.5%, 56.1%) of female high school students had poor menstrual hygiene management (MHM) practices. Having poor knowledge (AOR = 2.36, 95% CI: 1.43, 3.90), having lower attitude toward menses (AOR = 3.42, 95% CI: 2.10, 5.58), not having private shower at home (AOR = 2.37, 95% CI: 1.33, 4.20), not learning about menses at school (AOR = 1.8, 95% CI: 1.13,3.10), not having discussions about menses (AOR = 2.19, 95% CI: 1.26, 3.81), not having room for menstrual management at school (AOR = 2.06, 95% CI: 1.05, 4.05), and being grade 11‐12 (AOR = 0.53,95% CI: 0.33,0.85) were significantly associated with poor menstrual hygiene management practice.

**Conclusion:**

Over half of the students practice poor menstrual hygiene management, with key influencing factors being knowledge, attitude, facilities, education, and grade level. Therefore, efforts should focus on enhancing knowledge, changing attitudes, and improving facilities and resources for MHM in both homes and schools.

**Abbreviations**
AOR: adjusted odd ratio
CI: confidence interval
COR: crude odd ratio
LMIC: low‐ and middle‐income country
MHM: menstrual hygiene management
MOH: minister of health
NRR: non‐response rate
SPSS: statistical package for social science
SSA: Sub‐Saharan Africa
UNICEF: United Nations International Child, Emergency Fund
WaSH: water, sanitation and hygiene
WHO: World Health Organization.

## 1 Introduction

Menstrual hygiene management refers to the use of clean materials to absorb menstrual blood and changing them safely, privately, and hygienically as needed. It is a critical issue impacting health and psycho‐social outcomes, with schoolgirls being particularly vulnerable [1, 2].

According to the World Health Organization (WHO), 2.3 billion girls and women globally lack access to safe menstrual management, and approximately 113 million adolescent girls risk dropping out of school due to menstruation‐related challenges [3, 4]. Furthermore, Poor menstrual hygiene management (MHM) practices are linked to infections, reduced self‐esteem, and school absenteeism, common in developing countries that suffer from poverty and inadequate health care [5]. Studies from various countries have highlighted diverse menstrual hygiene management (MHM) practices and the factors influencing them, with prevalence rates varying significantly. For instance, poor MHM practices were reported among 33% of girls in Dang District, Nepal [6], 15.1% in West Gonja Municipality, Ghana [7], 46.9% in Bandung City, Indonesia [8], and 28.8% in Kajiado County, Kenya [9].

Despite global attention, the challenge remains highly relevant in Ethiopia, where regional disparities are notable. For instance, poor MHM practices were reported among 42.2% of girls in Harar City, Eastern Ethiopia [10], 51.1% in Dessie City, South Wollo Zone, Amhara Region [11], 49.97% in Motta Town, East Gojjam Zone, Amhara Region [12], and and 22% in Chelenko Town, East Hararghe Zone, Oromia Region [13]. These variations reflect differences in access to facilities, awareness, and cultural attitudes toward menstruation.

In response to the global need and to enhance safe menstrual hygiene practice, Menstrual Hygiene Day (MHD) is observed annually on May 28 to raise awareness about the importance of proper MHM in promoting dignity, health, and educational equity for girls and women [14]. Ethiopia has joined this global movement, with the government and partner organizations implementing programs aimed at improving access to menstrual hygiene products and education, particularly in schools. Additionally, international organizations have implemented programs to improve access to sanitation and hygiene services, with schools being a key focus [15]. Water, sanitation, and hygiene (WASH) programs in schools are also established to create a healthy environment and foster proper hygiene practices [16].

Despite national efforts to improve menstrual hygiene management (MHM) in Ethiopia, persistent challenges, including financial constraints, entrenched cultural taboos, geographic disparities, water and sanitation shortages, and uneven program implementation, continue to hinder progress [17]. Addressing these barriers is critical to safeguarding adolescent girls' health, dignity, education, and overall well‐being [18].

The Afar Region faces compounded challenges due to its pastoralist traditions, extreme climate, and remote geography. The pastoralist way of life limits access to essential infrastructure such as clean water, private and functional sanitation facilities, and affordable menstrual hygiene products [19]. Despite increasing national focus on improving MHM, there is limited evidence on menstrual hygiene practice and influencing factors in pastoralist regions like Afar. Therefore, this study aimed to assess menstrual hygiene management practices and associated factors among female high school students in the Awsi Rasu, Afar Region, Ethiopia.

## 2 Methods and Materials

### 2.1 Study Design, Period, and Setting

An institution‐based cross‐sectional study was conducted from April 15 to April 30, 2023, in secondary schools of Awsi Rasu Zone, Afar Region, northeastern Ethiopia.

Awsi Rasu is located approximately 356 km from Addis Ababa and shares borders with Gabi Rasu to the south, Hari Rasu to the southwest, the Amhara Region to the west, Fanti Rasu to the northwest, Kilbet Rasu to the north, Eritrea to the northeast, and Djibouti to the east. Administratively, the zone comprises 10 districts and two town administrations. The zone has 10 public secondary schools, serving a total of 12,976 students, of whom 5921 are female. The study area was purposefully selected due to its unique pastoralist culture, limited access to water, sanitation, and hygiene (WASH) services, and strong sociocultural norms affecting menstrual hygiene management (MHM) practices.

### 2.2 Source and Study Population

All female high school students attending regular school in Awsi Rasu were our source population, and these female high school students attending regular school during the data collection period were our study population.

### 2.3 Inclusion and Exclusion Criteria

Female students enrolled in selected high schools with a history of menstruation before data collection were included, while students unable to complete the tool due to illness were excluded.

### 2.4 Sample Size Determination

The sample size was calculated using a single population proportion formula with a 95% confidence level by considering a 48.8% proportion from a previous study of good menstrual hygiene management practices among schoolgirls in the north Wollo zone [11] with a tolerable error of 0.05. The formula used to calculate the sample size was as follows:

$$n=\frac{{\left(z\alpha /2\right)}^{2}p(1-p)}{d^{2}}=\frac{{\left(1.96\right)}^{2}0.488(1-0.488)}{0.05^{2}}=\approx 384,$$

where z = Z score for a 95% confidence interval, which is 1.96

*p* = expected prevalence or proportion of good menstrual hygiene management practices, which is 48.8% [11].

*d* = tolerable error between the sample and the true population, which is 5%.

After adding a 10% non‐response rate to the calculated sample size, 423 was taken for the final sample size.

### 2.5 Sampling Procedure and Technique

**Figure 1:**
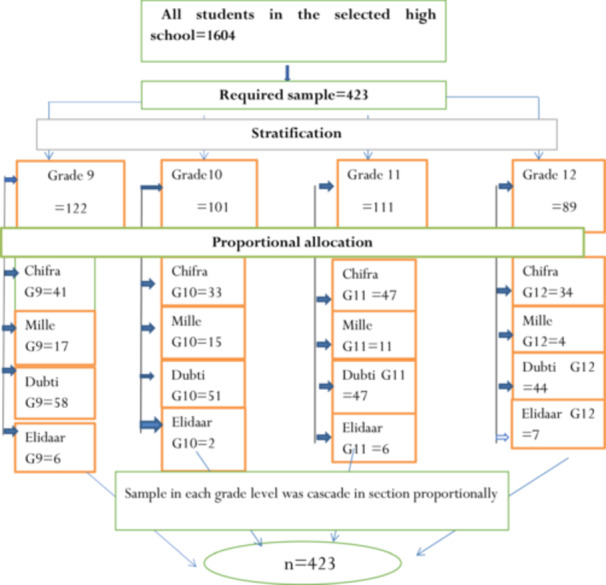
Schematic diagram showing the sampling technique and procedures to select a sample of 423 high school students at Awsi Rasu zone, afar region, Ethiopia, 2023.

The study population consisted of 1604 female students enrolled in four selected high schools: Chifra, Mille, Dubti, and Elidaar. These students were distributed across four grade levels, with 464 in Grade 9 (Chifra: 157, Mille: 64, Dubti: 219, Elidaar: 24), 382 in Grade 10 (Chifra: 124, Mille: 55, Dubti: 195, Elidaar: 8), 420 in Grade 11 (Chifra: 179, Mille: 42, Dubti: 177, Elidaar: 22), and 338 in Grade 12 (Chifra: 128, Mille: 15, Dubti: 168, Elidaar: 27). Since heterogeneity was assumed between grade levels, a stratified sampling method was applied with proportional allocation at each level of stratification. The calculated sample size of 423 students was proportionally distributed across the four grade levels based on their respective populations. Within each grade, the allocated sample was further stratified among the sections in proportion to their size. Finally, students from each section were selected using a simple random sampling method to ensure equal representation and fairness in selection (steps are described in Figure 1).

### 2.6 Study Variables

#### 2.6.1 Dependent Variable

The dependent variable in this study was menstrual hygiene management practice, categorized as good or poor.

#### 2.6.2 Independent Variable

Socio‐economic and demographic related variables: age, age at menarche, grade, marital status, religion, residence, parental education, parental occupation, and family monthly income.

Household and school‐related variables: pocket money, availability of a private shower and television at home, access to water and hygiene kits, learning about menstrual hygiene in school, hearing about menstruation before menarche, and discussing menstrual hygiene with others.

Knowledge and attitude‐related factors: Knowledge about menstruation and its management, as well as attitudes toward menstruation and its management.

### 2.7 Operational Definition

#### 2.7.1 Menstrual Hygiene Management Practice

To assess respondents' menstrual hygiene practices, 10 closed‐ended questions were used, with one point assigned for each correct response and zero for incorrect responses. The components of the assessment were as follows: (1) use of sanitary pad during menstruation (1 point for “yes,” 0 for "no"); (2) type of menstrual absorbent used (1 point for commercial or home‐made sanitary pads, 0 for underwear, sponge, or others); (3) washing of genitalia during menstruation (1 point for "yes," 0 for "no"); (4) frequency of genital washing (1 point for washing two or more times per day, 0 for less than two times); (5) material used for washing (1 point for soap and water or plain water, 0 for not washing); (6) bathing during menstruation (1 point for "yes," 0 for "no"); (7) frequency of bathing (1 point for bathing two or more times during menstruation, 0 for less than two times); (8) place of disposal of used menstrual absorbent (1 point for proper disposal such as in toilet pan, latrine, or with other waste, 0 for disposal in the open field); (9) frequency of changing sanitary materials (1 point for changing two or more times per day, 0 for less than two times); and (10) reuse of sanitary pad (1 point for “no,” 0 for “yes”). A total score was computed out of 10, and participants who scored five or more (≥ 5) were categorized as having good menstrual hygiene practices, while those who scored less than five (< 5) were considered to have poor practices [20, 21, 22].

#### 2.7.2 Knowledge on MHMP

Respondents' knowledge of menstrual hygiene was measured using 12 questions, with responses coded as 1 for correct answers and 0 for incorrect or ‘don't know’ responses.

#### 2.7.3 Good Knowledge

Respondents who scored ≥ 50% of the total knowledge questions.

#### 2.7.4 Poor Knowledge

Respondents who scored < 50% of the total knowledge questions [20].

#### 2.7.5 Attitudes Towards MHMP

Attitudes toward menstruation and menstrual hygiene were assessed using a four‐item Likert scale, with coding similar to that used for the knowledge questions.

Good attitude: Study participants who scored at or above the mean score of the respondent.

Poor attitude: Study participants who scored below the mean score of the respondent [23].

### 2.8 Data Collection Tool, Procedure, and Quality Control

A structured questionnaire was used after a review of relevant literature. Two experienced female teachers from each school, fluent in both Amharic and Afar, collected the data under the supervision of another female teacher. Data was collected using a structured self‐administered questionnaire.

To ensure data quality, the questionnaire was initially prepared in English, translated into Afaraf, and Amharic, and then back‐translated into English to ensure consistency. Before the actual data collection began, the questionnaire was pretested on 5% [22] of female students at Ali Mohammed Senior Secondary School, which was not included in the main study. Based on the pretest feedback, necessary revisions and refinements were made to the questionnaire. A supervisor was assigned to each school to oversee the data collection process, ensuring it was conducted properly and addressing any issues that arose. Additionally, a 1‐day training session was held for both data collectors and supervisors to familiarize them with the data collection tools and procedures.

### 2.9 Data Processing and Analysis

Data was checked, coded, and entered into EpiData (4.6) and was exported to SPSS V25 for further statistical analysis. A descriptive summary using frequency, percentage, mean, standard deviation, and proportions was conducted to describe the study population. The data were analyzed using bivariate and multivariable logistic regression to determine the effect of various factors on menstrual hygiene practice. All variables with a *p*‐value < 0.25 and below in the bivariable analysis were candidates for multivariable logistic regression, and then those variables with a *p*‐value < 0.05 in the multivariable analysis were declared as having a statistically significant association with menstrual hygiene management practice. To assess model fitness, the Hosmer‐Lemeshow goodness‐of‐fit test was performed (x² = 6.5866, df = 8, *p*‐value = 0.58), indicating a good model fit. Multicollinearity among independent variables was checked using the Variance Inflation Factor (VIF) and not detected (VIF < 10).

### 2.10 Ethics Approval and Consent to Participate

Ethical clearance for this study was obtained from the Institutional Review Board (IRB) of Samara University (Reference Number: ERC0010/2023). In addition, written permission was obtained from the Zonal Education Department and the principals of the selected schools.

Written informed consent was obtained from all participants aged 18 years and above. For participants under the age of 18, consent was obtained from their parents or legal guardians on their behalf, and assent was also obtained from the minors themselves. To ensure confidentiality, no personal identifiers were included in the questionnaire. Instead, unique code numbers were used, and all data were stored securely with password protection. The information collected was used solely for research purposes and was not misused or disclosed inappropriately. Participants were informed that their participation was entirely voluntary and that they had the right to withdraw from the study at any time without any consequences.

## 3 Results

### 3.1 Socio‐Demographic Characteristics of the Respondents

The data were collected from a total of 423 female high school adolescents, with a response rate of 100%. Nearly half of the participants were in the age group of 16–18 years, with a mean and SD of 17.13 and ±1.91 years, respectively. More than half of the study participants, 320 (75.7%), were urban residents (Table 1).

**Table 1:** Socio‐demographic characteristics of female high school students in Awsi Resu, Afar region, Ethiopia 2023 (*n* = 423).

Variable	Category	Frequency	Percentage (%)
Age	13–15	113	26.7
	16–18	202	47.8
	≥ 19	108	25.5
Age at menarche	13	151	35.7
	> 13	272	64.3
Grade level	9–10	223	52.7
	11–12	200	47.3
Resident	urban	320	75.7
	Rural	103	24.3
Ethnicity	Afar	227	53.7
	Amhara	151	35.7
	Tigray	33	7.8
	Other^a^	12	2.8
Religion	Muslim	348	82.3
	Orthodox	65	15.4
	Protestant	10	2.4
Marital status	Single	345	81.6
	Married	58	13.7
	Divorced	16	3.8
	Widowed	4	9.0
With whom do you live	Both parent	279	66.0
	Mother only	68	16.1
	Relatives	20	4.7
	Father only	14	3.3
	Husband	42	9.9
Educational status of the mother	No formal education	114	27.0
	Primary	167	39.5
	Secondary	80	18.9
	College and above	62	14.7
Educational status of father	No formal education	154	36.4
	Primary	174	41.1
	Secondary	61	14.4
	College and above	34	8.0
Family monthly income	≤ 3350	128	30.3
	3351–5000	117	27.7
	5001–8300	127	30.0
	≥ 8301	51	12.1

^a^ Oromia, SNNPR. Source of Information and Knowledge on Menstrual Hygiene.

Among female high school students, 85.1% received information about menstruation before menarche. Additionally, 69.5% of students reported discussing menstrual hygiene openly. While most students (88.4%) recognized menstruation as a physiological process, and 61.5% considered commercial sanitary pads comfortable, 34.8% had poor knowledge of menstrual hygiene management. Moreover, 55.8% mistakenly believed that girls could become pregnant during menstruation (Table 2).

**Table 2:** Pre‐menarche information sources, and knowledge about menstrual hygiene among female high school students in Awsi Resu, Afar Region, northeastern Ethiopia, 2023 (*n* = 423).

Variable	Category	Frequency	Percentage (%)
Did you hear about menses before menarche	Yes	360	85.1
	No	63	14.9
Discussion about menses	Yes	294	69.5
	No	129	30.5
Source of information (*n* = 360)	Mother	137	38.1
	Father	38	10.6
	Elder sister/s	71	19.7
	School (teacher, club, mini media	35	9.7
	Friend/peer	52	14.4
	Health professional	7	1.9
	Media	20	5.6
With whom (*N* = 294)	Mother	61	20.7
	Friends	138	46.9
	Teachers	40	13.6
	sisters	47	16
	Father	8	2.7
Menstruation	It is a physiological process	374	88.4
	It is a pathological process	11	2.6
	Curse from god	6	1.4
	I don't know	32	7.6
Cause of menstruation	Hormone	276	65.2
	It is caused by sin	11	2.6
	It is a curse of god	16	3.8
	It is caused by a disease	12	2.8
	I don't know	108	25.5
Source of menstruation	Uterus	300	70.9
	Vagina	42	9.9
	bladder	22	5.2
	Abdomen	26	6.1
	I don't know	33	7.8
How long is the normal bleeding duration	< 2 days	41	9.7
	2–7 days	338	79.9
	> 7 days	18	4.3
	I don't know	26	6.1
What is the normal duration of the menstrual cycle	< 21 days	87	20.6
	21–35 days	262	61.9
	> 35 days	26	6.1
	I don't know	48	11.3
You know, Poor menstrual hygiene predisposes to infection?	Yes	233	55.1
	No	190	44.9
During menses, frequently changing better to frequently change?	Yes	257	60.8
	No	166	39.2
Genitalia should be washed frequently during menses.	Yes	241	57
	No	182	43
Should girls take more nutrition during menstruation?	Yes	254	60
	No	169	40
Girl conceive during menstruation?	Yes	236	55.8
	No	187	44.2
Menstruation is life lifelong process?	Yes	187	44.2
	No	236	55.8
Good absorbent during menses	A commercial made a sanitary pad	260	61.5
	Homemade sanitary pad	37	8.7
	Under wear	103	24.3
	Sponge	23	5.4
Knowledge	Good	276	65.2
	Poor	147	34.8

### 3.2 Attitude Towards Menstrual Hygiene

In this study, 56.7% of female students demonstrated a low level of attitude toward menstrual hygiene management. For instance, 53.9% disagreed with the statement that poor menstrual hygiene is an unsafe practice that should be discouraged. Similarly, 56.7% disagreed that a lack of water is a contributing factor to poor menstrual hygiene practices (Table 3).

**Table 3:** Attitude towards menstrual hygiene among female high school students in Awsi Resu, Afar region, north‐east, Ethiopia 2023 (*n* = 423).

Variables	Strongly disagree	Disagree	Neutral	Agree	Strongly agree
Is a lack of water the reason for applying poor menstrual hygiene practices?	47.5%	9.2%	9.5%	15.6%	18.2%
Do you agree that poor menstrual hygiene is an unsafe practice and should be discouraged?	40.7%	13.2%	9.9%	17%	19.1%
Do you agree that using a public toilet feels uncomfortable for menstrual hygiene?	10.9%	19.6%	9.5%	35.7%	24.3%
Do you agree that most of the illnesses occur as a result of poor menstrual hygiene?	9%	8%	5.2%	31.9%	45.9%

### 3.3 Menstrual Hygiene Management Practice of the Respondent

According to the result of this study, the overall prevalence of good menstrual hygiene management practice among the respondents was 206 (48.7%), but more than half (217, 51.3%) of the respondents' menstrual hygiene management practice was poor. 371 (87.7%) of the students participating in the study use sanitary pads (Table 4).

**Table 4:** Menstrual hygiene management practice among female high school students in Awsi Resu, Afar region, north‐east, Ethiopia 2023 (*n* = 423).

Variable	Category	Frequency	Percentage
Use sanitary pad (*N* = 423)	Yes	371	87.7
	No	52	12.3
Material used for menstrual absorbent (*N* = 371)	A commercial made a sanitary pad	290	78.2
	Homemade sanitary pad	55	14.8
	underwear	20	5.4
	Sponge	6	1.6
Frequency of washing genitalia per day during menses (*N* = 423)	No	170	40.2
	One time	246	58.2
	Two time	2	0.5
	Three and more	5	1.2
material used for washing genitalia during menses (*N* = 423)	Soap and water	134	31.7
	Water only	119	28.1
	No	170	40.2
Take shower during menses (*N* = 423)	Yes	278	65.7
	No	145	34.3
Frequency of taking a shower during menses (*N* = 278)	One time	114	41.0
	Two time	58	20.9
	Three times	44	15.8
	Four or more times	62	22.3
Where were you disposed of used menstrual material (*N* = 423)	With other waste	70	16.5
	In the toilet pan	16	3.8
	In the open field	241	57
	In the latrine	96	22.7
How often do you change sanitary material per/day (*N* = 371)	One time	72	19.4
	Two time	86	23.2
	Three time	121	32.6
	Four or more times	92	24,8
Reuse a sanitary pad	Yes	189	50.9
	No	182	49.1
MHM practice	Good	206	48.7
	Poor	217	51.3

### 3.4 Household and School Characteristics of the Respondent

Half of the respondents earned pocket money. About 360 (85.1%) have room for managing menses at school. More than half, 268 (63.4%) of the respondents had a TV at home (Table 5).

**Table 5:** Household and school characteristics of female high school students in Awsi Resu, Afar region, north‐east, Ethiopia 2023 (*n* = 423).

Variable	Category	Frequency	Percentage
Earn permanent pocket money	Yes	211	49.9
	No	212	50.1
Feeling comfortable in school while menstruating	Yes	26	6.1
	No	397	93.9
Room for managing menses in school	Yes	360	85.1
	No	63	14.9
Learning menstrual hygiene in school	Yes	290	68.6
	No	133	31.4
Have access to clean water in school	Yes	337	79.7
	No	86	20.3
Have a hygienic kit (Dettol, cotton, soap) in school to use during menses	Yes	92	21.7
	No	331	78.3
Have a private shower at home	Yes	205	48.5
	No	218	51.5
Have a TV at home	Yes	268	63.4
	No	155	36.6

### 3.5 Factors Associated With Menstrual Hygiene Management Practice

A variable and multivariable logistic regression were used to identify variables associated with menstrual hygiene management practice. In the bivariate analysis, 15 variables are candidates for multivariate analysis. Place of residence, marital status, educational status of the mother, religion, grade level, knowledge of menses, attitude towards menses, age at menarche, availability of TV at home, availability of a private shower at home, availability of a room for menstrual hygiene management in school, learning about menstruation in school, availability of water in school, information about menstruation before menarche, and discussion about menstrual hygiene with others are associated with the practice of menstrual hygiene management. Seven of them are significantly associated with menstrual hygiene management practices.

High school students whose families do not have private showers at home were 2.37 times more likely to practice poor menstrual hygiene management as compared to students whose families have private showers at home (AOR = 2.37[1.33–4.20] 95% CI). On the other hand, students in grades 11–12 were 47% less likely to practice poor menstrual hygiene management than students in grades 9–10 (AOR = 0.53[0.33–0.85] 95% CI).

High school students with poor knowledge about menstruation and its management, 2.36 times, and a low attitude towards menstruation and its management were 3.42 times more likely to practice poor menstrual hygiene management as compared to their counterparts (AOR = 2.36 [1.43–3.89] 95% CI) and (AOR = 3.42 [2.10–5.58] 95% CI), respectively.

According to this study, adolescent girls who didn^’^t discuss menstrual hygiene and its management with others were 2.19 times more likely to have poor menstrual hygiene management practices than those who did discuss (AOR = 2.19 [1.26–3.81], 95% CI).

High school students who did not learn about menstruation and its management at school were 1.87 times more likely to have poor menstrual hygiene management practices compared to those who had received education on menstruation and its management at school (AOR = 1.87; 95% CI: 1.13–3.10). In addition to this, students who don't have room for managing menses at school were 2.06 times (adjusted OR = 2.06 [1.05–4.05] [95% CI]) more likely to have poor menstrual hygiene management practice than those who have room for managing menses at school (Table 6).

**Table 6:** Independent predictors of menstrual hygiene management practice among female high school students in Awsi Resu, Afar region, north‐east, Ethiopia 2023 (*n* = 423).

Variable	Response category	MHM Practice		COR (95% CI)	AOR (95%CI)	*p* value
		Poor	Good			
Grade level	9–10	131	92	1	1	
	11–12	86	114	**0.53 (0.36–0.78)**	**0.53 (0.33–0.85)**	**0.009** *
Have access to clean water in school	Yes	167	170	1	1	
	No	50	36	1.41 (0.88–2.28)	1.79 (0.98–3.28)	0.059
Attitude	Low	151	89	**3.01 (2.02–4.49)**	**3.42 (2.10–5.58)**	**0.000** *
	High	66	117	1	1	
Resident	Rural	68	35	2.23 (1.40–3.54)	0.81 (0.41–1.6)	0.544
	Urban	149	171	1	1	
Religion	Muslim	170	178	0.24 (0.5–1.14)	0.47 (0.67–3.27)	0.44
	Orthodox	39	26	0.64 (0.37–1.09)	0.49 (0.26–0.94)	0.032
	Protestant	8	2	1	1	
Knowledge	Poor	98	49	**2.64 (1.74–4.01)**	**2.36 (1.43–3.90)**	**0.001** *
	Good	119	157	1	1	
Marital status	Single	167	178	0.94 (0.13–6.74)	5.71 (0.57–69.2)	0.172
	Married	38	20	0.49 (0.28–0.88)	0.58 (0.29–1.16)	0.126
	Divorced	10	6	0.56 (0.2–1.58)	0.43 (0.11–1.60)	0.207
	Widowed	2	2	1	1	
Did you learn about menstrual hygiene in school	Yes	135	155	1	1	
	No	82	51	**1.85 (1.21–2.81)**	**1.87 (1.13–3.10)**	**0.016** *
Educational status of the mother	No formal education	60	54	1.76(0.94–3.30)	1.37(0.64–2.96)	0.414
	Primary	89	78	0.97(0.60–1.57)	0.73(0.41–1.29)	0.282
	secondary	44	36	0.91(0.51–1.61)	0.71(0.35–1.45)	0.351
	College and above	24	38	1	1	
Information before menarche	Yes	172	188	1	1	
	No	45	18	2.73 (1.52–4.9)	1.53 (0.68–3.42)	0.304
Discuss menstrual hygiene with others.	Yes	128	166	1	1	
	No	89	40	**2.89 (1.86–4.47)**	**2.19 (1.26–3.81)**	**0.005** *
Age at menarche	> 13	131	141	1	1	
	13	86	65	1.42 (0.95–2.13)	1.53 (0.94–2.48)	0.089
Room for managing menses in school	Yes	176	184	1	1	
	No	41	22	**1.95 (1.16–3.52)**	**2.06 (1.05–4.05)**	**0.036** *
Do you have a private shower at home	Yes	87	118	1	1	
	No	130	88	**2 (1.36–2.95)**	**2.37 (1.33–4.20)**	**0.003** *
Do you have a TV/radio at home?	Yes	131	137	1	1	
	No	86	69	1.30 (0.88–1.94)	1.09 (0.66–1.80)	0.726

*Note:* 1: Reference group.

* Significant association at *p*‐value < 0.05.

## 4 Discussion

Our study examined the prevalence of menstrual hygiene management practices and associated factors among female high school students in Awsirasu Afar, Ethiopia. The findings revealed that 51.3% (95% CI: 46.5%–56.1%) of the high school girls had poor menstrual hygiene management practices.

The findings of this study are similar to those of studies conducted in Bandung City, Indonesia (46.9%) [8], Dessie City, Amhara Region, Ethiopia (51.1%) [24], and Motta Town, Amhara Region, Ethiopia (49.97%) [12]. However, it is higher than findings reported in Dang District, Nepal (33%) [6], West Gonja Municipality, Ghana (15.1%) [7], Chitwan District, Nepal (27.5%) [25], Kajiado County, Kenya (28.8%) [9], Harar City, Eastern Ethiopia (42.2%) [26], Mekdela Woreda, Amhara Region, Ethiopia (37.6%) [27], and and Chelenko Town, Oromia Region, Ethiopia (22%) [13]. This disparity in Ethiopia could be attributed to differences in cultural openness regarding menstruation, better infrastructure in those areas, or targeted menstrual health interventions in those areas. In the context of Ethiopia, particularly in urban or semi‐urban areas, there may be greater availability of affordable menstrual products, better parent‐child communication, and stronger school engagement on MHM issues. In contrast, Awsi Rasu is a predominantly pastoralist area with limited access to WASH services, persistent menstrual taboos, and fewer community‐based initiatives targeting adolescent girls' health, which may contribute to the higher prevalence of poor practices. In contrast, the result of this study is lower than that of studies conducted in Gedeo Zone, Southern Nations, Nationalities, and Peoples' Region, Ethiopia (60.3%) [28], and Holeta Town, Oromia Region, Ethiopia (65.5%) [29]. The lower prevalence in our study may reflect recent efforts by stakeholders, such as local health authorities and NGOs, to increase awareness around menstrual hygiene. In the months leading up to the study, there were several school‐based educational activities and awareness campaigns in the study area, possibly improving girls' knowledge and practices compared to earlier or less‐targeted settings.

In this study, high school students whose families don't have private showers at home were 2.37 times more likely to practice poor menstrual hygiene management than students whose families have private showers at home. This finding is supported by a study undertaken in Mekdela City [27]. This is because water is an important thing to manage hygiene during menstruation [26].

In this study, students in grades 11–12 were 47% less likely to practice poor menstrual hygiene management than students in grades 9–10. This finding is supported by a study undertaken in Chelenko Town [13]. This may be because when girls' education levels increase, they gain more information about menstruation and how to manage it.

In this study, high school students with poor knowledge about menstruation and its management were 2.36 times more likely to practice poor menstrual hygiene management as compared to their counterparts. This finding is supported by different studies undertaken in Kenya [9], Gedio Zone [28], Harerge Zone [26], Mekdela City [27], North Wollo Zone [11], Gimbi Town [30], and Holeta Town [29]. It was supported by a systematic meta‐analysis study conducted in Ethiopia [31]. The girls' knowledge regarding menstruation and its management helps to overcome the negative effects of cultural beliefs and social taboos surrounding menstruation and its hygienic practices, as well as the community they live in. In addition to this, it might be that if they know what to do, it will be easier for them to practice properly.

In this study, students with a low attitude toward menstruation and its management were 3.42 times more likely to practice poor menstrual hygiene management as compared to their counterparts. This finding is supported by studies undertaken in Gedio Zone [28], Harerge [26], and the North Wollo Zone [11]. This might be because the attitude towards menstruation and its management is an important thing to know and practice.

According to this study, adolescent girls who didn't discuss menstrual hygiene with others were 2.19 times more likely to have poor menstrual hygiene management practices than those who did. This finding is supported by studies undertaken in Kenya [9], Addis Ababa [32], Dessie city [24], Boset district [33], and Gimbi Town [30]. This may be associated with the fact that girls who don't talk to their parents about their menstruation and its management know less about it, which discourages the practice of good menstrual hygiene management. This may be because girls who openly discuss menstruation and its management with parents or others are more likely to receive accurate information, guidance, and financial support, which enhances their ability to obtain sanitary pads and practice good menstrual hygiene management.

Poor menstrual hygiene management practices among high school students who didn^’^t learn about menstruation and its management at school were 1.87 times more likely than those who did. This finding is supported by studies undertaken in Addis Ababa [32] and Motsa town [12]. This might be due to the information provided about menstruation and its management at school, and it might be due to the fact that learning changes students' knowledge, attitude, and practice in menstrual hygiene management and enhances their commitment to practicing good menstrual hygiene management [31].

In this study, students who don't have room for managing menses at school are 2.06 times more likely to practice poor menstrual hygiene management than those who learn at school and have room for managing menses. This might be because infrastructure and supplies are important to manage menstruation properly, as they ensure a safe, private, and hygienic environment for girls and women. Access to adequate water, sanitation facilities, and menstrual hygiene materials allows individuals to change and dispose of sanitary products with dignity and maintain personal hygiene, which directly influences their menstrual hygiene management practices [5, 18, 26].

Despite the high proportion of sanitary pad use among participants (87.7%), the overall level of menstrual hygiene management practices remained low. This finding suggests that access to sanitary pads alone is insufficient to ensure safe and effective menstrual hygiene. Possible explanations include infrequent pad changing, inadequate genital hygiene due to limited access to water and soap, improper disposal practices, and insufficient knowledge of comprehensive menstrual hygiene management. These findings highlight the need for holistic interventions that extend beyond product availability to include education, adequate water and sanitation infrastructure, and social and familial support.

Furthermore, more than half of the respondents (57%) reported disposing of used menstrual pads in open fields, indicating significant gaps in menstrual waste management and awareness of safe disposal practices. Such improper disposal may contribute to environmental contamination, poor sanitation, and potential health risks, while also perpetuating stigma and secrecy surrounding menstruation. Similar challenges have been documented in other low‐resource settings, where inadequate school‐ and community‐level waste management systems hinder safe menstrual hygiene practices. This indicates the importance of integrated menstrual hygiene management interventions that combine access to sanitary materials with education on proper disposal methods and the provision of appropriate disposal facilities in schools and communities.

### 4.1 Limitations of the Study

Despite the strength of using a multi‐center approach, which helped collect data from different areas and made the findings more generalizable, this study had some limitations. Some questions asked about sensitive issues related to menstruation, which is often a taboo subject in the community. This could have led to social desirability bias, suggesting the need for future qualitative studies. There may also have been recall bias, especially for questions like remembering the age at first menstruation. In addition, there is a risk of selection bias because some girls who were menstruating might have been absent during data collection.

## 5 Conclusion

The study revealed that more than half of the respondents demonstrated poor menstrual hygiene management practices. Grade level, knowledge, and attitude towards menstruation, availability of a private shower at home, and a dedicated space for menstrual hygiene management in schools. Therefore, efforts to improve menstrual hygiene management should focus on enhancing knowledge and attitudes, improving WASH infrastructure in schools, and providing dedicated spaces for menstrual hygiene management to ensure adolescent girls' health and educational participation. Additionally, combining access to sanitary materials with education on proper disposal methods and the provision of appropriate disposal facilities in schools and communities is essential.

## Author Contributions

S.G., M.A., F.Y., A.Y., A.Y., H.A.Y., B.B.A., A.E.A., D.E.A. wrote the proposal, participated in data collection, analyzed the data, drafted the article, and prepared the article. They approved the proposal with a few revisions, participated in data analysis, and revised subsequent drafts of the article. All the authors read and approved the final article.

## Conflicts of Interest

The authors have declared that they have no competing interests.

## Transparency Statement

The lead author Degsew Ewunetie Anteneh affirms that this article is an honest, accurate, and transparent account of the study being reported; that no important aspects of the study have been omitted; and that any discrepancies from the study as planned (and, if relevant, registered) have been explained.

## Data Availability

The dataset analyzed in this study is available from the corresponding author and can be accessed as a supporting file.
